# Species-specific evolution of immune receptor tyrosine based activation motif-containing CEACAM1-related immune receptors in the dog

**DOI:** 10.1186/1471-2148-7-196

**Published:** 2007-10-18

**Authors:** Robert Kammerer, Tanja Popp, Stefan Härtle, Bernhard B Singer, Wolfgang Zimmermann

**Affiliations:** 1 Tumor Immunology Laboratory, LIFE Center, Klinikum Grosshadern, Ludwig-Maximilians-University, Marchioninistr. 23, 81377 Munich, Germany; 2 Institute for Molecular Immunology, GSF National Research Center for the Environment and Health, Marchioninistr. 25, 81377 Munich, Germany; 3 Institute for Animal Physiology, Ludwig-Maximilians-University, D-80539 Munich, Germany; 4 Institute for Anatomy, University Hospital Essen, Hufelandstr. 55, 45147 Essen, Germany

## Abstract

**Background:**

Although the impact of pathogens on the evolution of the mammalian immune system is still under debate, proteins, which both regulate immune responses and serve as cellular receptors for pathogens should be at the forefront of pathogen-driven host evolution. The CEA (carcinoembryonic antigen) gene family codes for such proteins and indeed shows tremendous species-specific variation between human and rodents. Since little is known about the CEA gene family in other lineages of placental mammals, we expected to gain new insights into the evolution of the rapidly diverging CEA family by analyzing the CEA family of the dog.

**Results:**

Here we describe the complete CEA gene family in the dog. We found that the gene coding for the ITIM-bearing immunoregulatory molecule CEACAM1 gave rise to a recent expansion of the canine CEA gene family by gene duplication, similar to that previously found in humans and mice. However, while the murine and human CEACAMs (carcinoembryonic antigen-related cell adhesion molecules) are predominantly secreted and GPI-anchored, respectively, in the dog, most of the CEACAMs represent ITAM-bearing transmembrane proteins. One of these proteins, CEACAM28, exhibits nearly complete sequence identity with the ligand-binding N domain of CEACAM1, but antagonizing signaling motifs in the cytoplasmic tail. Comparison of nonsynonymous and synonymous substitutions indicates that the CEACAM28 N domain is under the strongest purifying selection of all canine CEACAM1-related CEACAMs. In addition, CEACAM28 shows a similar expression pattern in resting immune cells and tissues as CEACAM1. However, upon activation CEACAM28 mRNA and CEACAM1 mRNA are differentially regulated.

**Conclusion:**

Thus, CEACAM1 and CEACAM28 are the first paired immune receptors identified within the CEA gene family, which are expressed on T cells and are most likely involved in the fine-tuning of T cell responses. The direction of gene conversion accompanied by purifying selection and expression in immune cells suggests the possibility that CEACAM28 evolved in response to selective pressure imposed by species-specific pathogens.

## Background

The evolution of immunoglobulin superfamily (IgSF) members is largely influenced by the nature of their ligands. The extracellular part of IgSF members which are predominantly expressed in the nervous system are well conserved, while the extracellular domains of members expressed by immune cells or cells involved in reproduction tend to diversify much more rapidly [1]. The CEA gene family, which belongs to the IgSF, is a tandemly clustered multigene family. Such gene families are subject to rapid evolution due to ongoing gene duplication, deletion and mutational events [2]. At present, the CEA family is subdivided into two main subgroups: the CEA-related cell adhesion molecule (CEACAM) and the pregnancy-specific glycoprotein (PSG) subgroups. The CEACAM subgroup in humans consists of 12 members composed of a single immunoglobulin variable (IgV)-like N-terminal (N) domain followed by zero to six Ig constant (IgC)-like domains of A and B subtypes and one member which consists of two IgC-like domains and two IgV-like domains, one at each end of the molecule. CEACAM1, CEACAM3, CEACAM4, CEACAM18, CEACAM19, CEACAM20 and CEACAM21 are transmembrane molecules while CEA/CEACAM5, CEACAM6, CEACAM7, and CEACAM8 are linked to the cell membrane via glycosylphosphatidylinositol (GPI) anchors and CEACAM16 is most likely a secreted molecule. For CEACAM1 (also known as CD66a, BGP and C-CAM105) and the recently discovered CEACAM16, CEACAM18, CEACAM19 and CEACAM20, but not for other family members have orthologs been identified in rodents[3]. In addition to these orthologs, the transmembrane-bound CEACAM2 (in mice), CEACAM17 (in mice and rats) and the secreted members CEACAM9, CEACAM10, CEACAM11 CEACAM12, CEACAM13, CEACAM14, CEACAM15 (in mice and rats) exist in rodents[3,4]. In mice, rats and cattle, at least two CEACAM1 alleles each have been identified, which differ considerably in their N domain sequences[5]. No such allelic variation has been observed in humans. The PSG, which are specifically expressed in the trophoblast, are the most abundant fetal proteins in the maternal bloodstream during late human pregnancy. The PSGs are thought to play a pivotal role in the regulation of the maternal immune response to the fetal semi-allograft[6]. The diversity of the CEACAM/PSG family of proteins is further enhanced by species-specific differential splicing of several family members[7]. As expected from the variable structure of the CEA family members, these molecules have diverse functions. It is well established that various CEA family members play crucial roles in cell-cell adhesion, tumor development, angiogenesis, insulin metabolism, reproduction and, as more recently realized, in immunity[8]. CEACAM1 functions as a natural killer cell inhibitory receptor[9,10], regulates T and B cell proliferation [11,12], induces dendritic cell maturation[13] and facilitates granulocyte and monocyte survival [14,15]. In addition, CEACAM3, CEACAM6 and CEACAM8 were found to be pivotal for the regulation of granulocyte activation in humans[16]. While human CEACAM1 contains two immunoreceptor tyrosine-based inhibitory motifs (ITIM) in its cytoplasmic domain, one CEACAM1 ITIM is replaced by an immunoreceptor tyrosine-based switch motif (ITSM) in mice and rats. Human CEACAM3 and possibly CEACAM4, CEACAM19 and CEACAM20 harbor functional immunoreceptor tyrosine-based activation motifs (ITAM) in their cytoplasmic tails [17,18]. In contrast, except for CEACAM19 and CEACAM20 no such ITAM-containing CEA family members exist in rodents. Remarkably, several pathogens namely, *Neisseria sp*., *Haemophilus influenzae*, and *Moraxella catarrhalis *in humans[19-21] and mouse hepatitis virus (MHV) [22] use CEACAMs to anchor themselves to or to invade host cells. In addition, it has been suggested that bacterial pathogens can down-regulate immune functions by binding to CEACAM1 on CD4+ T lymphocytes and signaling via the CEACAM1 ITIM motif [23]. It was recently proposed that the human granulocyte-specific CEACAM3 functions as a specifically adapted single-chain ITAM-dependent phagocytic receptor involved in the clearance of CEACAM-binding bacteria by human granulocytes[24]. This indicates that fixation of genes in the human genome coding for ITAM-bearing CEACAMs can be the result of a selective pressure mediated by CEACAM-binding pathogenic microbes. In contrast, one of the two allelic variants of murine CEACAM1 exhibits a significant lower virus receptor activity suggesting that, in mice, mutations in the CEACAM1 gene rather than the fixation of an activating virus receptor were acquired to evade pathogen attack. The availability of a high-quality draft genome sequence of the domestic dog (*Canis familiaris*) [25] provided the possibility to analyze the complete CEA gene family in a third mammalian order. Here we show that in the dog multiple ITAM-bearing CEACAM1-related CEACAMs evolved and that CEACAM28 and CEACAM1 have undergone concerted evolution by gene conversion. Based on sequence analyses and comparison of expression profiles we suggest that CEACAM28 and CEACAM1 are paired activating and inhibitory immune receptors involved in the regulation of T cell responses. The possibility that sporadic recurrence of selective pressure mediated by species-specific pathogens was the driving force for the evolution of multiple ITAM-bearing CEACAM1-related CEACAMs in the dog is discussed.

## Results

### Identification of the canine CEA gene family cluster

**Figure 1:**
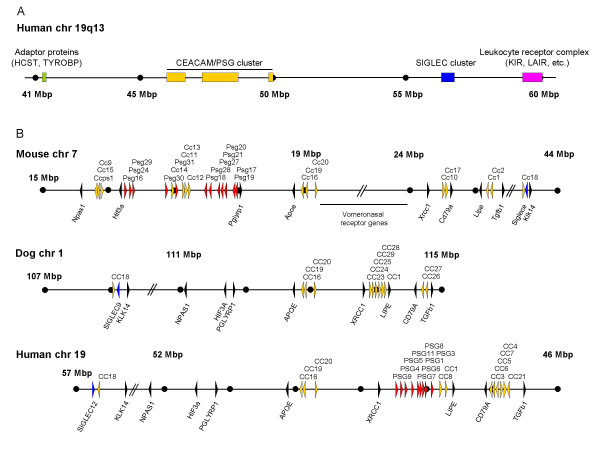
**Genomic arrangement of the human extended Leukocyte Receptor Complex and syntenic relationship of the CEACAM and PSG loci in human, mouse and dog**. (A) The location and size of the genomic regions on human chromosome 19q13 containing genes for immune adaptor proteins, CEACAM/PSGs, SIGLEC proteins and the Leukocyte Receptor Complex are indicated by colored bars. (B) Genomic organization of mouse, dog and human CEA gene family loci. Arrowheads represent genes with their transcriptional orientation. CEACAM genes are shown in yellow, PSG genes in red, SIGLEC genes in blue and marker genes in black. The scale indicated by dots is 1 Mbp unless interrupted by slanted lines. The following Ensembl/NCBI releases were used: mouse, NCBIm36; dog, CanFam 1.0, WGS database; human, NCBI 36. Nucleotide numbering of the chromosomes starts at the centromere. Note the inverse orientation of the human chromosome 19 region. CC/Cc, CEACAM/Ceacam; chr, chromosome.

In an effort to identify all CEA gene family members in the dog genome we employed the BLAST program to search the GenBank and Ensemble databases (CanFam 1.0, July 2004) using cDNA nucleotide sequences from all known human and mouse CEA family members as query sequences. The human and mouse CEA gene families are located within the expanded leukocyte receptor complex on chromosome 19q13 (Figure 1A) and chromosome 7, respectively. This approach yielded the sequences of the CEACAM1, CEACAM16, CEACAM18, CEACAM19 and CEACAM20 orthologs. Their predicted full length mRNA sequences (Additional file 1) encode proteins with domain organizations identical with their human and murine counterparts: CEACAM16 (N1-A-B-N2); CEACAM18 (N-A-B-TM-Cyt); CEACAM19 (N-TM-Cyt); CEACAM20 (truncated N-A1-B1-A2-B2-TM-Cyt)[3]. Like their mouse and human counterparts, both CEACAM19 and CEACAM20 contain presumed ITAM consensus sequences in their cytoplasmic domains. However, like in mouse, no CEACAM19 cytoplasmic domain exon 1 present in human CEACAM19 was found [3]. As for human CEACAM18, no cytoplasmic domain exon could be identified due to its small size and lack of corresponding EST sequences. In a second round of search we used the BLAST program and cDNA nucleotide sequences from the identified orthologous canine genes to screen the GenBank and Ensemble databases. This strategy was employed in order to identify dog-specific genes which have evolved after the separation of the dog and primate/rodent lineage. This approach led to the identification of 7 additional CEACAM genes (*CEACAM23-CEACAM29*) using CEACAM1 nucleotide sequences as query sequences. *CEACAM23, CEACAM24, CEACAM25, CEACAM29, CEACAM28 *and *CEACAM1 *are arranged between *XRCC1 *and *LIPE*, while *CEACAM26 *and *CEACAM27 *are located between *CD79A *and *TGFβ1 *on the opposite strand (Figure 1). Surprisingly, no genes corresponding to the *PSG *genes were found. The murine *Psg *genes are located between *Hif3a *and *Pglyrp1 *spanning nearly 1.8 Mbp. In dog, however, the distance between *HIF3A *and *PGLYRP1 *comprises only 0.1 Mbp which rules out the presence of a larger set of genes between these marker genes. Similarly, there is not enough space between *XRCC1 *and *CEACAM23 *(0.2 Mbp) to accommodate an expanded *PSG *gene family as found in humans at the corresponding chromosomal locations location where the distance is approximately 1 Mbp (Figure 1B). The canine CEACAM gene loci on dog chromosome 1 exhibit a similar arrangement to that found for the human CEA gene family on chromosome 19 but it is partially different from that of the murine CEA gene family on chromosome 7 (Figure 1B).

### Identification of exons coding for the cytoplasmic domain of CEACAM1-related CEACAMs

**Figure 2:**
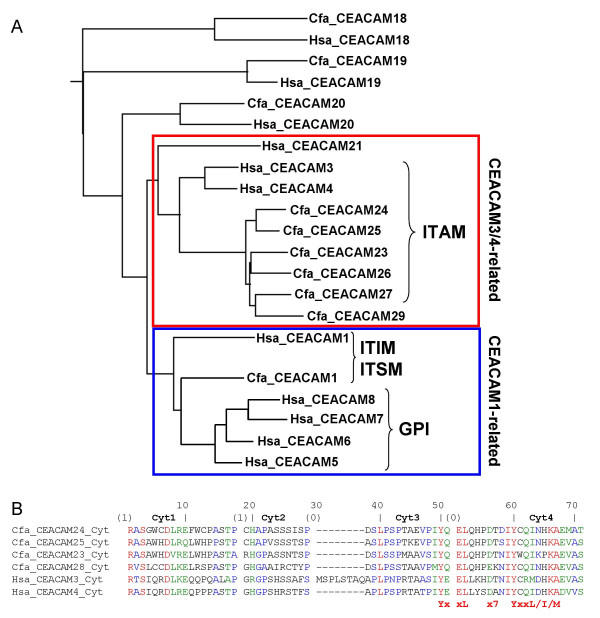
**Expansion of ITAM-bearing CEACAM1-related CEA family members in dog**. (A) Relationship of transmembrane domain and hydrophobic GPI signal peptide-encoding exon sequences of human and canine CEA family members. The nucleotide sequences of the transmembrane domain exons and exons encoding the GPI signal peptides of human CEA family members were aligned and the results displayed as rooted dendrogram. Three groups can be discriminated: the transmembrane domain sequences of the orthologous genes (CEACAM18, CEACAM19 and CEACAM20) form pairs, CEACAM3, CEACAM4 and CEACAM21 sequences are clustered together with the dog CEACAM1-related sequences (red box) and the GPI signal sequences are most closely related to the CEACAM1 transmembrane exon sequence (blue box). All transmembrane domain exons boxed red are followed by exons encoding cytoplasmic domains with an ITAM except for CEACAM21 and CEACAM29. Their cytoplasmic domains terminate prematurely due to a stop codon at the end of cytoplasmic domain exon 2 and loss of the splice donor site in cytoplasmic domain exon 3 with subsequent read-through into the following intron, respectively. (B) Amino acid sequence alignment of cytoplasmic domains (encoded by the cytoplasmic domain exons Cyt1-Cyt4) from dog CEACAM1-related proteins. The ITAM consensus sequence is indicated below and the intron phases above the sequences. Note the typical split by a phase 0 intron of the first YxxL motif of the ITAM motifs. Hsa, *Homo sapiens*; Cfa, *Canis familiaris*.

**Figure 3:**
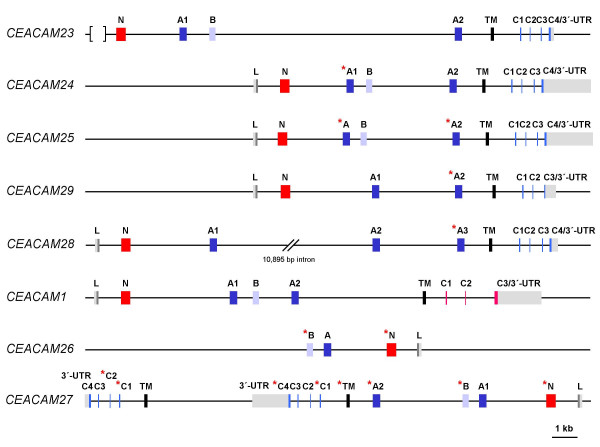
**Exon arrangement of canine CEACAM1-related genes**. The exon types are indicated by differently colored boxes. 5'- and 3'-UTR are shown as gray, IgV-like domain exons as red, IgC-like domain exons as blue and transmembrane domain exons as black boxes. The exons encoding the cytoplasmic domain with an ITIM or an ITAM motif are shown in red and blue, respectively. The size of the 3'-UTR is inferred from the position of the first putative polyadenylation signal sequence (AATAAA) after the stop codon. The presence of deletions/insertions in exons causing reading frame shifts or mutational corrupted splice donor and acceptor consensus sequences are indicated by an asterisk. No leader exon could be identified for CEACAM23 because of a sequence gap (indicated by brackets) in the publicly available genomic sequences. The genes are arranged in the order and orientation as found on dog chromosome 1.

In order to identify the very short cytoplasmic exons of the CEACAM1-like genes which cannot be localized by direct search strategies we first localized the transmembrane exons of all CEACAM genes using transmembrane exon sequences of the human and mouse CEACAM genes as query sequences. Surprisingly, the 6 predicted transmembrane domain exons of the CEACAM1-related canine genes clustered with the sequences of the transmembrane domain exons of human CEACAM3, CEACAM4 and CEACAM21 which are connected to cytoplasmic domain exons encoding a C-terminal ITAM motif (CEACAM3 and CEACAM4) or a homologous truncated cytoplasmic domain with a premature stop (CEACAM21). The sequence of the transmembrane exon of canine CEACAM1 clustered with transmembrane sequences from murine Ceacam1 and Ceacam2 and with a group of exons coding for hydrophobic GPI signal peptides from human CEACAM1, CEACAM5, CEACAM6, CEACAM7, CEACAM8 (Figure 2A and data not shown). CEACAM5-CEACAM8 GPI signal peptide exons are thought to have evolved from the transmembrane exon of an ancestral CEACAM1 by introduction of a stop codon [26]. CEACAM1/Ceacam1 and Ceacam2 encode cytoplasmic domains which contain ITIM motifs. Taken together, this indicates that TM and cytoplasmic domain exons are exchanged together during evolution and predicts the presence of ITAM signaling motifs rather than ITIM in the cytoplasmic tails of the dog CEACAM23-CEACAM25, CEACAM28 and CEACAM29 molecules. Subsequently, we screened about 2,000 bp of nucleotide sequence downstream of the predicted canine transmembrane domain exons for the presence of the short cytoplasmic exons. Indeed, small cytoplasmic domain exons similar to the ones found in human CEACAM3 and CEACAM4 could be identified, which encode ITAM motifs close to the C-terminal end (Figure 2B). Although homologous cytoplasmic domain exons 1–3 are present in CEACAM29 the loss of the splice donor site in the cytoplasmic domain exon 3 leads to read through into the adjacent intron and truncation by a stop codon following after five codons. CEACAM26 and CEACAM27 probably represent pseudogenes due to multiple deletions in their extracellular (in particular N exons) and cytoplasmic domain exons (Figure 3).

### Relationship of the canine primordial CEACAM genes with human and murine counterparts

To determine the degree of relatedness between the primordial canine CEACAM genes with the orthologous genes in mice and man, the nucleotide and encoded amino acid sequences of the leader, the IgV-type N domain, the IgC-type A and B domains, the transmembrane domain and the cytoplasmic domain exons were compared (Table 1). The degree of sequence identity between corresponding extracellular Ig-type domains in dog, humans and mice varies greatly at the amino acid sequence level. The lowest value is observed for CEACAM1 N domains (56% and 42% for human and mouse, respectively), the highest for the CEACAM16 N2 domain (92 and 96% for human and mouse, respectively; Table 1).

**Table 1:** Relationship between dog CEA family members and human and mouse orthologs

	**ortholog**	**L**	**N1**	**A1**	**B1**	**A2**	**B2**	**N2**	**TM**	**Cyt**
**CEACAM1**	human	64 (47)	73 (56)	79 (68)	83 (76)	75 (60)			63 (48)	79 (61)
	mouse	57 (47)	62 (42)	74 (64)	80 (66)	71 (58)			67 (64)	78 (65)
**CEACAM16**	human	86 (83)	88 (89)	89 (91)	88 (91)			91 (92)		
	mouse	83 (58)	83 (88)	83 (87)	81 (89)			88 (96)		
**CEACAM18**	human	76 (58)	77 (60)	72 (57)	73 (62)				69 (52)	
	mouse	46 (47)	71 (66)	65 (55)	68 (60)				51 (35)	
**CEACAM19**	human	83 (66)	82 (78)						81 (69)	78 (65)
	mouse	51 (41)	75 (68)						76 (59)	75 (59)
**CEACAM20**	human	86 (76)	79 (57)	78 (62)	79 (68)	76 (65)	77 (70)		72 (56)	65 (41)
	mouse	59 (23)	65 (35)	69 (53)	71 (54)	72 (64)	72 (66)		66 (51)	56 (40)

The nucleotide and amino acid sequences of leader (L), IgV-like domain (N1, N2), IgC-like domain (A1, A2, B1, B2), transmembrane domain (TM) and combined cytoplasmic domain exons (Cyt) were compared pairwise using the ClustalW software (Weight Matrix BLOSOM). The degree of identity is indicated in % (amino acid sequence identity in brackets).

### Cloning of full-length canine CEACAM1-related cDNAs and identification of splice variants

**Figure 4:**
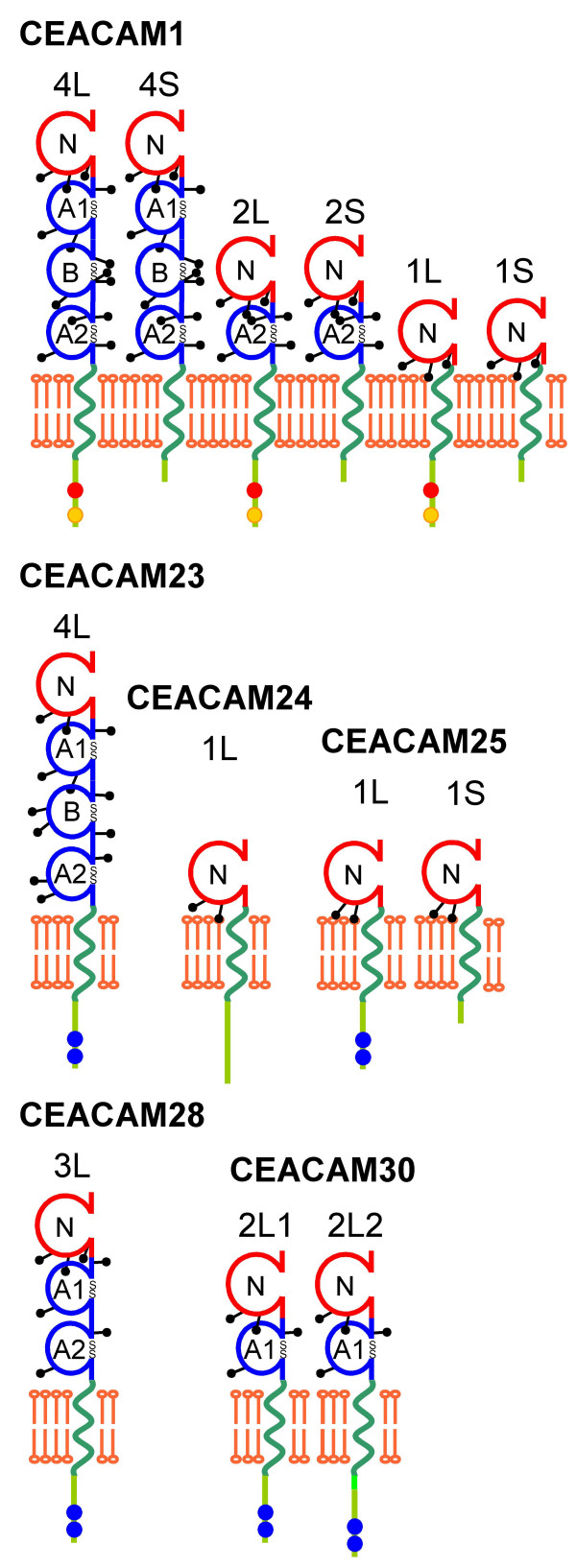
**Domain organization of dog CEACAM1-related protein splice variants**. The domain organization has been predicted by data mining based on genomic databases and was confirmed by RT-PCR amplification, cDNA cloning and sequencing. The number of Ig-related domains and the presence of either a long (L) or short cytoplasmic domain (S) are indicated above the schematic representation of the proteins. Potential signal consensus motifs in the cytoplasmic domains are indicated as red (ITIM), yellow (ITSM) and blue dots (ITAM). Potential N-glycosylation sites are indicated by lollipops, disulfide bridges by SS.

Based on the genomic sequence information we designed gene-specific primers for the amplification of full length CEACAM1-related cDNAs from total RNA, isolated either from liver or spleen. In the published genomic dog sequence, one nucleotide is missing from the A1 exon of canine *CEACAM1*. Cloning and sequencing of full length CEACAM1 clones from a mix-breed dog revealed a complete 279 bp CEACAM1 A1 exon. In total, we could identify 6 different CEACAM1 splice variants. The largest clone contains an open reading frame of 1563 bp coding for a 521 amino acid protein. Comparisons with CEACAM1 from other species identified the cloned canine CEA family member unequivocally as CEACAM1. It is composed of a leader sequence, four extracellular immunoglobulin-like domains (N, A1, B and A2), a transmembrane domain and a cytoplasmic domain (Figure 4). The cytoplasmic domain contains an N-terminal ITIM and a C-terminal ITSM motif similar to that found in the long CEACAM1 isoforms of mouse, rat and cattle. According to the nomenclature system for the CEA family, the longest canine CEACAM1 splice variant was designated CEACAM1-4L (GenBank accession no. DQ975208). The mRNA encoding the splice variant with the short cytoplasmic tail CEACAM1-4S (GenBank accession no. DQ975209) differs from CEACAM1-4L mRNA by the absence of the 53 bp nucleotide cytoplasmic domain exon1 that leads to a frame shift and to the use of a TGA stop codon in cytoplasmic domain exon 2 as found for *CEACAM1 *of other species. Splice variants without the A1 and the B domain designated CEACAM1-2L (GenBank accession no. DQ975210) and CEACAM1-2S (GenBank accession no. DQ975211) also exist as well as two splice variants without IgC-like domains [CEACAM1-1L (GenBank accession no. DQ975212) and CEACAM1-1S (GenBank accession no. DQ975213)]. Furthermore, we have cloned the most abundant full length CEACAM23, CEACAM24, CEACAM25, and CEACAM28 cDNAs. The CEACAM23 cDNA (GenBank accession no. EF137906) corresponds to the longest mRNA splice variant predicted from the genomic sequence of the boxer breed and exhibits an identical nucleotide sequence. CEACAM23 encodes four extracellular Ig-like domains, a transmembrane domain and a cytoplasmic tail which harbors a typical ITAM motif (N-A1-B-A2-TM-Cyt1-Cyt4; Figure 4). The major CEACAM24 cDNA encodes a protein with an N and a transmembrane domain (GenBank accession no. EF137907). The cytoplasmic domain does not contain the expected ITAM motif due to the presence of the unspliced intron between the predicted cytoplasmic domain exons 3 and 4. The cloned CEACAM25 splice variants also code for proteins with only one N domain followed by a transmembrane domain. Three out of four clones encode cytoplasmic domains which contain the predicted ITAM motif (GenBank accession no. EF137908). In one clone, the absence of the 53 nucleotide cytoplasmic domain exon 1 leads to a frame shift and the usage of an alternative stop codon located in cytoplasmic domain exon3 (GenBank accession no. EF137909). Using supposedly CEACAM28-specific primers, two products were amplified which differ in their length by 276 bp. Cloning and sequencing revealed that the CEACAM28 gene codes for a protein with one N domain, two A domains, a transmembrane domain and a cytoplasmic tail which contain an ITAM motif (GenBank accession no. EF137910). The sequence of the cDNAs cloned from the smaller PCR product was different from all genomic sequences published either for the boxer or the poodle breed and, therefore, was named CEACAM30. The differences are more or less randomly distributed within the whole coding region which consists of an N, A, transmembrane and cytoplasmic domain region. Two CEACAM30 splice variants were identified, both containing an ITAM motif in the cytoplasmic tail. They differ, however, in the usage of alternative splice acceptor sites of the cytoplasmic domain exon 1 (GenBank accession nos. EF137911 and EF137912) (Figure 4).

### Relationship of CEACAM1-like genes and CEACAM1

**Figure 5:**
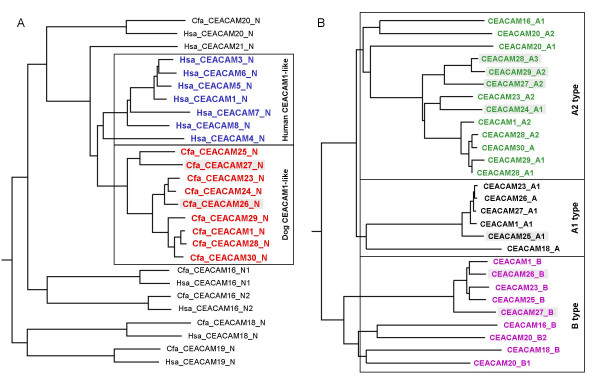
**Relationships of dog CEA gene family members**. N domain exon nucleotide sequences from all CEACAM members of the human and dog CEA gene families (A) or dog CEACAM exons encoding IgC-like domains (B) were aligned and the results depicted as rooted dendrograms. Human and dog N domain exon sequences from CEACAM1-related genes are shown in blue and red, the ones from other CEACAM genes in black. The CEACAM1-like gene IgC type domain B, A1 and A2 type nucleotide sequences are depicted in magenta, dark and light green, respectively. Sequences from presumed pseudogenes are marked by shading. Note that the primordial N exon sequences are most closely related with their respective orthologous counterpart sequences (except for the primate-specific CEACAM21), while the human and canine CEACAM1-related N exon sequences cluster with that of members of the same species. Interestingly, CEACAM28 contains two CEACAM1 A2 type domains (A1, A2) which has not yet been observed for other CEA family members.

**Figure 6:**
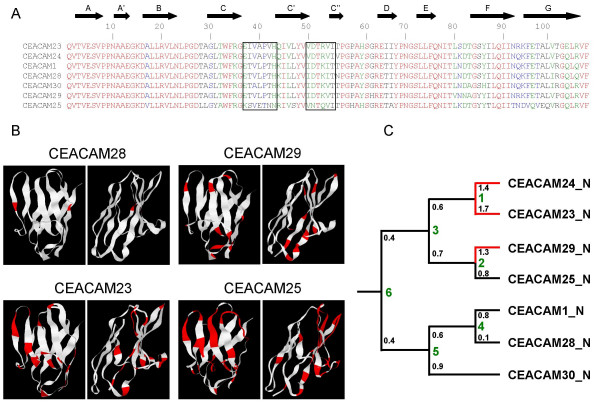
**Relationships of the N-domains of dog CEA gene family members**. (A) The mature N domain amino acid sequences of the dog CEACAM1-related CEA family members were aligned using the ClustalW program provided at the NPS server. Identical amino acids are shown in red, conservatively exchanged amino acids in blue and green and non-conservative changes in black. (B) The amino acid sequence positions which differ between CEACAM1 and the CEACAM1-related N domains (shown in red) are mapped to 3-D ribbon models calculated using the Geno3D-release 2 program. The models are shown with the CFG sheet in front (left model) and facing to the left (right model). (C) The ratios of non-synonymous and synonymous mutation in the N domain exons (small black numbers at the branches of the dendrogram) are close to one or higher (except for the nearly identical CEACAM1 and CEACAM28 sequences). This indicates selective pressure for divergence of N domain sequences during evolution. Hsa, *Homo sapiens*; Cfa, *Canis familiaris*.

To analyze the evolutionary relationship of the newly discovered CEACAM genes, multiple nucleotide sequence alignments of Ig domain exons of human and dog CEACAM genes were performed and phylogenetic trees constructed. As expected, the N domain sequences of the orthologous genes (*CEACAM16, CEACAM18-CEACAM20*) clustered pairwise, while all new canine N domain exon sequences (*CEACAM23-CEACAM30*) clustered with that from dog *CEACAM1 *(Figure 5A). Similarly, the primate-specific CEACAM1-related N domain nucleotide sequences (*CEACAM3-CEACAM8*) clustered with the human *CEACAM1 *N domain exon sequence except for *CEACAM21 *N. The close relationship between the new dog CEACAM genes and dog *CEACAM1 *could also be demonstrated when the A and B domain exon nucleotide sequences were compared (Figure 5B). Nine of the 17 IgC-like domains of the new CEACAM1-related canine molecules are of the CEACAM1 A2 type and five each were of the CEACAM1 A1 and B type (Figure 5B).*CEACAM23 *appears to be the only *CEACAM1*-related gene which contains all extracellular domain exons present in *CEACAM1*, in particular three IgC-like domain exons (A1, A2 and B). All other members of this subgroup either lack one of these exons or contain exons corrupted by nonsense, frame shift or splice site mutations leading to smaller predicted proteins (Figure 3, 4). Assuming that the N domain is the most relevant domain for ligand interaction, the ligand specificity of CEACAM28 seems to be most closely related to that of CEACAM1. The N domain amino acid sequence of CEACAM28 differs from that of CEACAM1 in only two positions. These amino acid changes do not affect the center of the CFG face, a topological N domain region of CEACAM members essential for ligand and pathogen interactions (Figure 6A,B). Interestingly, *CEACAM28 *lies next to *CEACAM1 *in the *CEACAM1 *gene cluster which hints to an evolutionary recent duplication event (Figure 1B). *CEACAM25 *is the most distant *CEACAM1 *relative within the group of *CEACAM1*-related genes, sharing only 66% of its N domain exon encoded amino acid sequence (Figure 5A, 6A; Table 2). Most of the amino acid sequence differences cluster to the CFG face of the N domains, indicating a positive selection for ligand diversification. To test this possibility, we calculated the ratio of nonsynonymous (Ka) to synonymous (Ks) nucleotide substitution rates on the branches of an N exon phylogenetic tree. The lowest Ka/Ks ratio (0.1) is observed for CEACAM28. This indicates that the CEACAM28 N domain is under purifying selection. For all other canine CEACAM1-related CEACAMs, the N domain amino acid changes appear to evolve neutrally or to be positively selected for during evolution since the corresponding Ka/Ks values are close to 1 or higher (Figure 6C).

**Table 2:** Relationship of dog CEACAM1-related family members

**A N**	**CEACAM1**	**CEACAM23**	**CEACAM24**	**CEACAM25**	**CEACAM28**	**CEACAM29**	**CEACAM30**
**CEACAM1**		90	90	79	98	93	95
**CEACAM23**	83		96	80	89	89	88
**CEACAM24**	85	94		80	90	89	89
**CEACAM25**	66	71	71		78	79	78
**CEACAM28**	98	81	84	64		93	96
**CEACAM29**	89	83	84	66	87		95
**CEACAM30**	93	82	84	65	93	90	

The amino acid (A; below the diagonal) and nucleotide sequences (N; above the diagonal) of IgV-like domain exons were compared pairwise using the ClustalW software (Weight Matrix BLOSOM). The degree of identity is indicated in %.

### ITAM-bearing CEACAM1-related genes are expressed by cells of the immune system

**Figure 7:**
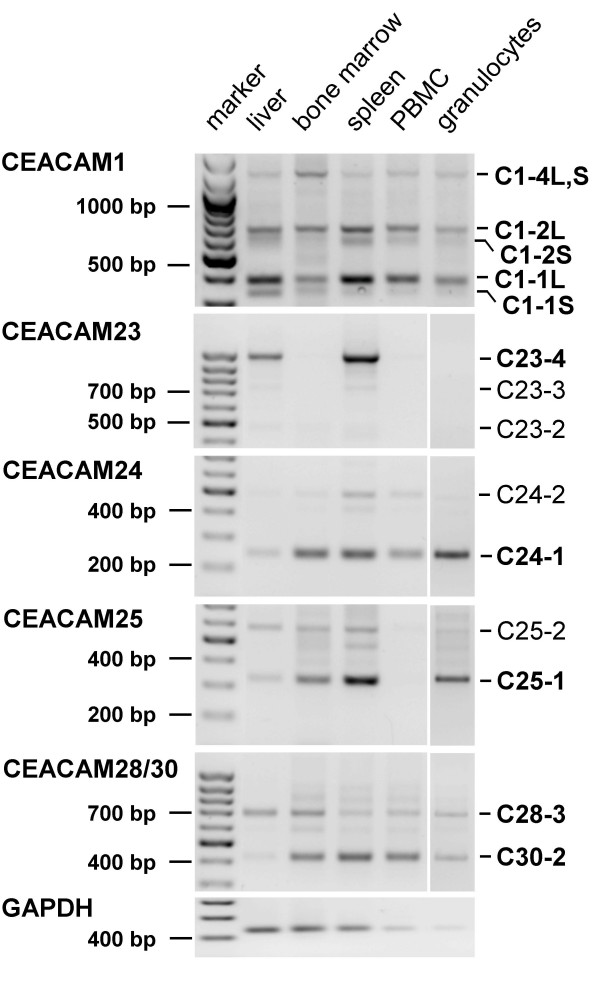
**Expression pattern of dog CEACAM1-related genes**. CEACAM1-related transcripts were identified by RT-PCR using gene-specific primers which are located in the N domain and transmembrane exons. For the detection of CEACAM1 transcripts, primers in the N domain and cytoplasmic domain exon 3 were used. The products were separated by agarose gel electrophoresis in the presence of ethidium bromide and visualized by UV illumination. One-kb and 100-bp DNA fragment ladders were used as markers. The possible domain organization of the proteins encoded by the splice variants (number of Ig domains) is indicated in the right margin. Sequence determination of the CEACAM28 PCR products revealed simultaneous detection of CEACAM28 and CEACAM30 cDNAs (till then unknown). C, CEACAM.

To identify the CEACAM gene expression pattern and splice variants, their nucleotide sequences were aligned, and regions of the transmembrane domain-encoding exons with sequence differences were selected to design primers that are specific for each of the CEACAM1-related cDNAs except for CEACAM28 and CEACAM30 which were amplified with a common primer pair (Table 3). The primers were used to amplify cDNAs from individual CEACAM by RT-PCR from RNA isolated from liver, bone marrow, spleen, purified peripheral blood mononuclear cells (PBMC), and granulocytes (Figure 7). Except *CEACAM29*, all analyzed *CEACAM1*-related genes, (*CEACAM1, CEACAM23, CEACAM24, CEACAM25, CEACAM28, and CEACAM30*) were found to be expressed in spleen. CEACAM1, CEACAM24, CEACAM25, CEACAM28 and CEACAM30 transcripts were detected in bone marrow and CEACAM1, CEACAM24, CEACAM28 and CEACAM30 mRNAs in PBMC. Granulocytes express in addition to different CEACAM1 splice variants (preferentially with the long cytoplasmic tail) all the ITAM-bearing molecules except CEACAM23. In liver, the presence of mainly CEACAM1 and CEACAM23 transcripts could be demonstrated. *CEACAM26 *and *CEACAM27 *are not expected to be expressed because the sequences of essential exons appear to be corrupted (see above).

**Table 3:** Gene-specific oligonucleotides for cloning and expression analyses of dog *CEA *family members

**gene**	**oligonucleotide name**	**oligonucleotide sequence**	**location of primers (exon)**	size of PCR product (bp)*
*CEACAM1*	cfCEACAM1-5i-f cfCEACAM1-3i-r	5'-CGGCAGCTGCTCTCACC 5'-CCAGCAGGACAGGTTGCAT	5'-UTR 3'-UTR	1624
	CEACAM1cf-5i CEACAM1cf-3i	5'-GTCATGAAGCTTCGGCAGCTGCTCTCACC 5'-GTCAGTTCTAGACCAGCAGGACAGGTTGCAT	5'-UTR 3'-UTR	1648
	cfCEACAM1TM-f cfCEACAM1C3-r	5'-AGTTCTGGCCTTCCACCTG 5'-GGATGAGGAGGCTGAAGTTG	TM domain Cyt 3	270
	cfCEACAM1N-f cfCEACAM1C3-r	5'-CATCACCCTGAACGACACTG 5'-GGATGAGGAGGCTGAAGTTG	N domain Cyt 3	1183
*CEACAM23*	cfaCC23-N-fo cfaCC23-T-re	5'-TCCAGAACATCACCCTGAGC 5'-GACACCCCAGGGAAGCTG	N domain TM domain	1008
	cfCEACAM1-5i-f cfaCC23-3-re	5'-CGGCAGCTGCTCTCACC 5'-GAGCAGTACCATGGGGAAAC	5'-UTR 3'-UTR	1616
*CEACAM24*	cfaCC24-N-fo cfaCC24-T-re	5'-GTAGTAGACACACGAGTAATTGT 5'-CACAAGCGCTACTCCAATCA	N domain TM domain	258
	cfCEACAM1-5i-f cfaCC24-3-re	5'-CGGCAGCTGCTCTCACC 5'-GAGGGTTCCAGGGTGAAACT	5'-UTR 3'-UTR	1069
*CEACAM25*	cfaCC25-N-fo cfaCC25-T-re	5'-AATCTGCCTGGGGATCTTCT 5'-AATACGCCAGGGTAGCCATG	N domain TM domain	352
	cfCEACAM1-5i-f cfaCC25-3-re	5'-CGGCAGCTGCTCTCACC 5'-GGAAGGAGGGTTCCAAGGT	5'-UTR 3'-UTR	807
CEACAM28 (CEACAM30)**	cfaCC28-N-fo cfaCC28-T-re	5'-TTTGAAACTGCACTAATACGG 5'-TTGTGTGGAGCAGGAGACA	N domain TM domain	703 (427)
	cfCEACAM1-5i-f cfaCC28-3-re	5'-CGGCAGCTGCTCTCACC 5'-GGAAGGAGAAGTGTCATGAGGT	5'-UTR 3'-UTR	1374 (1095)
*CEACAM29*	cfaCC29-N-fo cfaCC29-T-re	5'-ACATCACCGTGAATAATGCC 5'-CGAGTGCTAGTTCGAGCAGA	N domain TM domain	447
	cfCEACAM1-5i-f cfaCC29-3-re	5'-CGGCAGCTGCTCTCACC 5'-CAGAAGGTTACAGGGGCTCA	5'-UTR 3'-UTR	844

* Size of largest cloned or predicted (CEACAM29) splice variant is shown

** CEACAM30 cDNA were also detected using the CEACAM28 primers (size of PCR products in brackets)

Cyt, cytoplasmic domain; TM, transmembrane domain; UTR, untranslated region

### Expression of CEACAM1 and CEACAM28 by T cells upon activation

**Figure 8:**
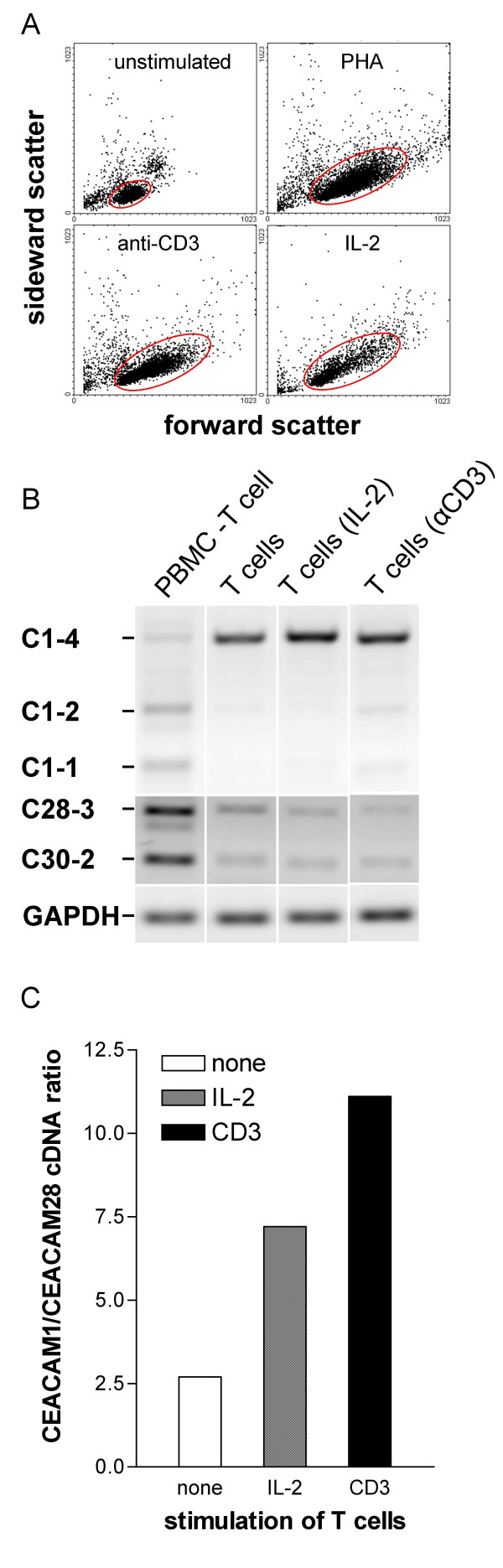
**Expression of the paired receptors CEACAM1 and CEACAM28 in stimulated T cells**. RNA was isolated from purified lymphocyte populations. CEACAM1 and CEACAM28 transcripts were identified by RT-PCR. Gene-specific primers located in the N domain and transmembrane exons were used for CEACAM1 cDNA amplification. The primers for CEACAM28 cDNA detection coamplified CEACAM30 cDNA due the close relatedness of the two genes. For comparison, GAPDH cDNA (226 bp) was amplified from the same cDNA samples. (A) Activation of T cells was controlled by determining blast formation by flow cytometry. Blast formation characterized by cell enlargement is demonstrated by an increase of the forward scatter of the T cells (encircled cell populations) (B) The products were separated by agarose gel electrophoresis in the presence of ethidium bromide and visualized by UV illumination. The amount of cDNA was quantified by endpoint determination with the Quantity 1^® ^software. Note the increase of the CEACAM1/CEACAM28 cDNA ratio after stimulation of T cells with IL-2 and CD3 (C).

To further analyze the expression of CEACAM1-related CEACAMs by immune cells, we isolated T cells from PBMC by MACS sorting using CD4- and CD8-specific mAbs. This procedure resulted in a purity of the T cell populations of >97%, as determined by flow cytometry (data not shown). Activation of T cells by the various stimuli was controlled by analysis of blast formation using flow cytometry (Figure 8A). RT-PCR analysis demonstrated that resting T cells express CEACAM1, CEACAM28 and CEACAM30 mRNA simultaneously. In addition, expression of these CEACAMs was also found in T cell-depleted PBMC, which are mainly composed of Bcells (Figure 8B). Stimulation of PBMC with anti-CD3 mAb or IL-2 had only a minor effect on CEACAM1 expression in T cell-depleted PBMC (data not shown) and T cells. In contrast, the content of CEACAM28 mRNA seem to be reduced in T cells upon activation (Figure 8B). In unstimulated T cells the CEACAM1/CEACAM28 cDNA ratio was < 3 whereas the ratio was > 7 and > 11 in T cells stimulated with IL-2 and anti-CD3 mAb, respectively (Figure 8C). The content of CEACAM30 mRNA remained unaltered in T cells upon stimulation (Figure 8B).

### CEACAM1 and CEACAM28 are paired immune receptors created by gene conversion

**Figure 9:**
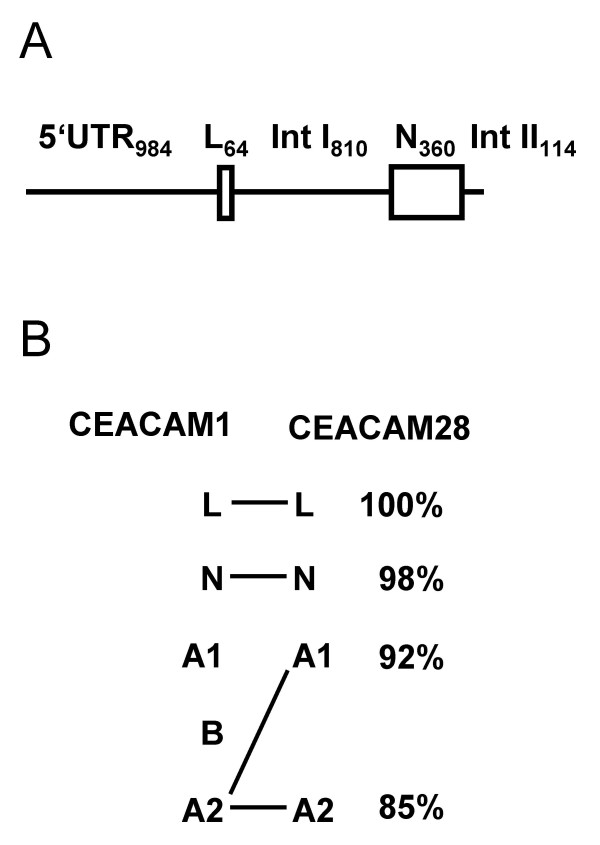
**CEACAM1 and CEACAM28 genomic regions involved in gene conversion**. The 2332 bp genomic region of CEACAM1 which covers nearly 1 kb of the 5'-flanking region (5'-FR), the leader exon (L), intron 1 (IntI), the N domain exon (N) and part of intron 2 (Int II) is highly conserved in *CEACAM28 *(99%) and, therefore, probably participated in a recent gene conversion event. The homologous sequences upstream and downstream from the conserved region are less conserved (80% and 92%, respectively) (A). This can also be deduced from the degree of conservation of the amino acid sequences encoded by the leader, N and A domains which is much less outside of the gene conversion region. Note that CEACAM28 contains two A2 type domains (B).

The near identity of the N domains between CEACAM1 and CEACAM28 is most likely due to a recent gene conversion event. Indeed, we could identify a 2,332 bp genomic region in *CEACAM1 *which shows 99% identity with the corresponding region of *CEACAM28*. The high similarity of this segment starts at position -984 of the 5'-flanking region and ends at position 114 of the intron between the Ndomain and the A1 domain exons (Figure 9A). Upstream of this segment, the sequence similarity between homologous regions of CEACAM28 and CEACAM1 (~1,200 bp) drops to 80%. The downstream homologous region of ~1,700 bp demarcated by a SINE element in CEACAM28 exhibits a similarity of 92%. The occurrence of such a regionally restricted gene conversion event is also supported by the high degree of amino acid sequence similarity of the leader (100%) and N domain (98%). The A1 and A2 domains of CEACAM28 encoded outside of the conserved genomic region share only 88% and 86% of their amino acid sequence with that of the homologous CEACAM1 A2 domain (Figure 9B). These data suggests that the similarity between *CEACAM1 *and the *CEACAM28 *ancestor was ~92% before the gene conversion event occurred.

## Discussion

Recent molecular studies have established strong evidence for four primary, superordinal mammalian clades: Afrotheria, Xenarthra, Euarchontoglires and Laurasiatheria [27,28]. The CEA gene family is well known in primates (humans) and rodents (mice and rats). However, both orders belong to the same principal lineage of placental mammals, the Euarchontoglires. Since carnivores belong to the Laurasiatheria clade, we expected to gain new insights into the evolution of the rapidly diverging CEA family by analysis of the CEA family of the dog. First of all, identification of *CEACAM1, CEACAM16, CEACAM18, CEACAM19 *and *CEACAM20 *orthologs in the dog genome at corresponding chromosomal locations, strongly indicates that the CEA gene family of the common ancestor of Euarchontoglires and Laurasiatheria consisted of at least five genes. It remains to be seen whether species which represent older mammalian lineages like Afrotheria, marsupials and monotremes contain the same set of primordial genes. Furthermore, the findings of this work supports our previous hypothesis that *CEACAM3-CEACAM8, CEACAM21 *and *CEACAM9-CEACAM15, CEACAM17*, none of which is present in the dog genome (Figure 2A and data not shown), represent primate and rodent-specific genes, respectively [3]. The ancestral members of the CEA gene family are located in three different loci (Figure 1). While *CEACAM16*, *CEACAM19 *and *CEACAM20 *are close together (*CEACAM16 *and *CEACAM19 *are next to each other), *CEACAM1 *is separated from this locus by more than 1 Mbp and *CEACAM18 *is displaced into the *SIGLEC *gene cluster in humans, mice and dogs.

Out of these ancestral genes, probably only *CEACAM1 *ancestors gave rise to massive gene expansions in a order-specific manner [3]. The *CEACAM1*-related genes form compact clusters next to *CEACAM1 *and near the *CD79A *gene. In rodents even a third locus (around the *Hif3a *gene) has been encroached by amplified *CEACAM1*-related genes. With analysis of the CEA family in a third order (carnivores), the inversion event observed for the chromosomal region flanked by *Cd79a *and *Lipe *can now be decided to have happened in the rodent rather than in the primate lineage, since the order of genes in this locus is conserved between humans and dogs (Figure 1).

*CEACAM1*-related genes can be subdivided into the *PSG *and the CEACAM subgroup genes. The birth of the ancestral *PSG *has been estimated to have occurred some 90 Myr ago [29], approximately the time of rodent-primate divergence[30]. The rapid, independent expansion of the human and mouse *PSG *gene families occurred through further gene duplication and exon shuffling events [31-33]. Surprisingly, we did not find any *PSG *gene in the dog genome indicating that the PSG function exerted in primates and rodents is dispensable for the dog, possibly because dogs have an endotheliochorial placenta type which is different from the hemochorial placentae of primates and rodents[34]. Semi-allogeneic fetal trophoblast cells are less invasive and concomitantly less prone to encounter the maternal immune system in endotheliochorial placentae in comparison to the hemochorial placentae where the trophoblast cells are bathed in maternal blood[35]. Therefore, PSG could be involved in the regulation of trophoblast invasion and/or modulation of the maternal immune system as has been suggest before[3,36]. Alternatively, the function of the *PSG *could have been taken over by an unrelated gene family due to functional convergence at the molecular level, similar to that found for natural killer (NK) cell receptors, where killer cell immunoglobulin-like receptors (KIR) and C-type lectin-like receptors (Ly49) are used in primates and rodents, respectively.

In contrast to the PSG, the CEACAM subgroup of canine *CEACAM1*-related genes underwent a recent expansion similar to that found in primates and rodents. In general, IgSF members which are expressed by leucocytes are diverging rapidly within their extracellular domains. This results in an average amino acid identity of about 54% between human and mouse orthologs [1]. The conservation of the CEACAM1 N domain, most relevant for pathogen and cell-cell interactions [37], lies within this range, with an amino acid identity of 56% (dog versus human) and 42% (dog versus mouse). This is consistent with the notion that CEACAM1 plays a key role in various functions of the immune system[8]. In contrast, between mouse and human CEACAM16, CEACAM18 and CEACAM19 orthologs, the N domain amino acid identities are higher ranging from 60% to 91%, which argues for non-immunological functions, especially for CEACAM16, which exhibits an overall amino acid sequence identity of about 90% (Table 1).

The dog CEACAM1-related genes are arranged head to tail, like the KIR genes in the human KIR locus[38,39]. Such an arrangement was found to facilitate unequal crossing over leading to gene expansion and generation of multigene families [40]. Furthermore, the KIR gene family is subject to birth-and-death evolution, domain shuffling and mutational changes [41]. Our analyses indicate that the CEA gene family evolved similarly. One characteristic for this kind of evolution is the presence of multiple pseudogenes, which also exist within the canine CEACAM1-like gene locus (CEACAM26, CEACAM27).

This type of evolution also facilitates a mechanism for qualitatively changing a receptor's signaling potential. A number of type I transmembrane IgSF members including CEACAMs and KIRs are composed of an amino-terminal extracellular "environmental sensor" and a carboxy-terminal cytoplasmic tail which can contain either inhibitory (ITIM) or activating motifs (ITAM or a positively charged amino acid in the transmembrane region permitting coupling with activating adaptors such as DAP12[42]). Unequal crossing-over at such gene loci encompassing the corresponding exons could result, in a single step, in genes encoding receptors with a drastically changed signaling potential by combining the same extracellular domain with a cytoplasmic tail of opposite function. This allows rapid adaptation to environmental changes and the creation of so-called paired immune receptors. An example for such a mechanism is found for the KIR gene KIR3DL/S, where either an inhibitory variant or an activating variant encoding KIR gene is found in different human individuals at the same locus[43]. In addition, a change in the receptor's signaling potential which may be the result of an arms race between pathogens and the host immune system has been reported recently for mice. The murine cytomegalovirus (MCMV) induces in infected cells expression of the virus-encoded GPI-anchored m157 protein, which binds to the activating, DAP12-associating killer receptor Ly49H in MCMV-resistant mice leading to NKcell-mediated cytotoxicity. MCMV-susceptible mouse strains do not have Ly49H but the extracellularly very similar ITIM-bearing Ly49I protein which has inhibitory killer cell receptor activity. Binding of m157 to LY49I in such mice may inhibit NK cell cytotoxicity thus rendering the mice susceptible to MCMV infection[44]. It was suggested that the activating receptor gene was derived from the gene encoding the inhibitory receptor to counteract a virus that exploits the inhibitory receptor during infection and that this mechanism may apply to many other activating receptors that have inhibitory counterparts[45,46]. However, recently it was shown for the human *SIGLEC5 *and *SIGLEC14 *genes that the direction of gene conversion that generates paired immune receptors is not always the same. This indicates that also other needs drive the evolution of paired receptors like the fine tuning of immune responses [45]. Host-pathogen interactions involving both proteins with ITIM or ITAM signaling motifs have also been reported for humans. The primate-specific CEACAM1-related protein CEACAM3 which contains an N domain closely related to that of CEACAM1 (88% amino acid sequence identity) and a cytoplasmic tail with an ITAM is exclusively expressed in granulocytes. Due to its close relatedness with the ITIM-bearing gonococcal receptor CEACAM1 it can act as decoy receptor for Neisserial pathogens and is able to induce clearance of gonococcal infections by granulocytes. Mutational analyses demonstrated that the cytoplasmic ITAM is instrumental for this process [24].

To our knowledge, CEACAM1 and CEACAM28 represent the first paired coexpressed immune receptors which contain antagonizing ITIM and ITAM (and not just a transmembrane domain with a positively charged amino acid which serves as a docking site for ITAM-containing adaptor molecules, like DAP12) in their respective cytoplasmic tails. CEACAM28 was probably formed from a CEACAM29 ancestor by a recent gene conversion encompassing the leader and the N exon from CEACAM1. The latter exon encodes the region most instrumental for ligand and pathogen binding in CEACAM molecules. Several findings, presented here, argue for a role of CEACAM28 as a CEACAM1 decoy receptor in the dog. First, the direction of the gene conversion was most likely from the inhibitory receptor to the activating receptor, second, the Ka/Ks ratio of the N domain exons of these family members is indicative for a purifying selection, whereby the specificity of the putative CEACAM1 ligand is probably conserved for CEACAM28, third, the amino acid changes observed for the CEACAM28 N domain most likely have no effect on the structure of that part of the N domain shown to be the pathogen binding site in human and mouse CEACAM1 and fourth, is expressed by immune cells (T, probably B and/or NK cells and granulocytes), a prerequisite for a defense function against a putative pathogen. Based on a highly similar amino acid sequence and expression pattern CEACAM30 can be envisioned to also function, like CEACAM28, as a decoy receptor.

However, this hypothesis raises questions about the driving force behind the fixation of the other ITAM-bearing CEACAM1-related genes in the dog. Most likely, selective pressure by pathogens changes with time. In times where the selective pressure exerted by pathogens on a receptor system is low, Darwinian selection will act and change the specificity of a receptor now possibly recognizing an endogenous ligand. Once the receptor has gained a new function it also can change the signaling properties which may explain why older i.e. more diverged ITAM-bearing receptors e.g. like CEACAM24 and CEACAM29 have lost the ITAM or have gained new splice variants which do not contain an ITAM.

## Conclusion

Taken together, the presence of multiple CEACAM1-related dog CEACAMs with ITAMs could reflect a repeated arms race between species-specific pathogens and the dog immune system. Fixation and expression by effector cells of ITAM-containing CEACAMs would result from either bacterial pathogens binding to CEACAM1 or viruses which induce the expression of CEACAM1 ligands by infected cells as found for MCMV. This putatively pathogen-driven evolution of the CEA gene family led to the development of the CEACAM1/CEACAM28 receptor pair which could also be involved in the fine tuning of T cell responses.

## Methods

### Cells and tissues

Different canine tissue samples were collected from healthy mix-bread dogs and flash-frozen in liquid nitrogen. Peripheral blood mononuclear cells (PBMCs) and granulocytes were isolated from blood of healthy mix-bread or beagle dogs by density-gradient centrifugation through Ficoll-Paque (GE Healthcare, Freiburg, Germany). Granulocytes located on top of erythrocytes were harvested and remaining erythrocytes were lysed (ammonium chloride buffer).

Stimulation of PBMC with PHA (2 μg/ml for 72 hr), CD3 (CA17-2A12;[47]) (0,5 μg/ml for 96 hr) and human IL-2 (200 U/ml for 7 days) was performed at a concentration of 5 × 10^5 ^cells/ml in RPMI-1640 supplemented with 10% fetal calf serum (FCS "Gold"; PAA Laboratories, Coelbe, Germany), 2 mM L-glutamine, 100 U/ml penicillin, 100 μg/ml streptomycin, non-essential amino acids and 1 mM sodium pyruvate (GIBCO/Invitrogen, Karlsruhe, Germany). T cells were isolated from either stimulated or unstimulated PBMC by positive MACS sorting following the manufacturer's instructions (Miltenyi Biotec, Bergisch-Gladbach, Germany). Stimulated and unstimulated PBMC were incubated with mouse anti-canine CD4 (CA13-1) and rat anti-canine CD8 mAbs (Dog 10-8)[48] and subsequently with bead-coupled anti-mouse Fc mAb which also reacts with rat IgG.

### Flow cytometric analyses

For surface staining, cells were suspended in PBS/0.3% (w/v) BSA supplemented with 0.1% (w/v) sodium azide. Cells were incubated with 0.5 μg/10^6 ^cells of the relevant mAb for 30 min at 4°C. Cells were washed twice and analyzed with a FACScan (BD Biosciences, Mountain View, CA). Dead cells were excluded by propidium iodide staining. The following reagents and mAbs from Serotec were used: FITC-conjugated anti-CD3, FITC-conjugated anti-CD4 and PE-conjugated anti-CD8α.

### Reverse transcription-polymerase chain reaction analysis

Total RNA extraction was performed using either the TRIzol^® ^reagent (Invitrogen Life Technologies, Karlsruhe, Germany) or the RNeasy Kit (Qiagen, Hilden, Germany) according to the manufactures' protocols. One μg of total RNA was used for cDNA synthesis by reverse transcription (RT) in a total volume of 20 μl using either the Reverse Transcription System^® ^(Promega, Mannheim, Germany) or the First-Strand cDNA Synthesis^® ^Kit (MBI Fermentas, St. Leon-Rot, Germany). The RT product (1 μl) was amplified by polymerase chain reaction (PCR) with Taq polymerase (Qiagen). After an initial denaturation step at 95°C for 45 s, 35 PCR cycles (denaturation: 95°C, 30 s; annealing: 60°C, 1 min; extension: 72°C, 1.5 min) and a final extension step at 72°C for 15 min were performed. The primers were designed using the Primer3 software. Gene-specific primers were selected that exhibited > 2 mismatches at the 3'-end of the oligonucleotide to all other members of the canine CEACAM family, except for CEACAM28 and CEACAM30 (see below). The primers were validated by eye using multiple nucleotide sequence alignments of the aforementioned CEA family members. The primer sequence and the predicted sizes of the amplified products are summarized in Table 3. Canine GAPDH cDNA was amplified using GAPDH.dogforward 5'-GCCAAAAGGGTCATCATCTC and GAPDH.dog reverse 5'-GCCCATCCACAGTCTTCT primers at a annealing temperature of 56°C and 30 cycles. Eight μl of each PCR were analyzed by electrophoresis on a 1.8 % agarose gel and visualized by ethidium bromide staining. The amount of cDNA was quantified by endpoint determination with the Quantity 1^® ^software (Bio-Rad Laboratories, Munich, Germany).

### Cloning and sequence determination of dog CEACAM1 and related cDNAs

Based on published genomic sequences and comparison with human *CEACAM1 *the 5'- and the 3'-untranslated regions of canine CEACAM1 were predicted and used to design primers for amplification of full length cDNA (CEACAM1cf-5i and CEACAM1cf-3i; Table 3). These primers introduced *Hin*dIII and *Xba*I restriction sites at the 5'- and 3'-ends of the PCR products, respectively. PCR was performed with cDNA from dog liver using *Pfu *DNA polymerase according to the protocol supplied by the manufacturer. After electrophoretic separation, 6 distinct ethidium bromide-stained DNA fragments were excised from the agarose gel and purified using the Perfectprep^® ^Gel Cleanup Kit (Eppendorf, Hamburg, Germany). The full length cDNAs were digested with *Hind*III and *Xba*I and cloned into the pRc/CMV expression vector. Plasmid DNA isolated from 12 clones were analyzed by PCR and sequencing. Full length cDNAs of CEACAM23, CEACAM24, CEACAM25, CEACAM28 and CEACAM30 were cloned using the TOPO TA Cloning^® ^Kit (Invitrogen Life Technologies). PCR was performed with cDNA from dog spleen using Taq polymerase (Qiagen) and the CfCEACAM1-5i-f forward primer for all CEACAMs combined with gene-specific reverse primers from the 3'-untranslated regions (UTR; Table 3).

### Biocomputing

Sequence similarity searches were performed using the NCBI BLAST tools [49] and Ensembl BLAST/SSAHA search programs[50]. For the identification of CEACAM genes not yet annotated in the dog genome, sequences from human and mouse CEACAM and PSG cDNAs were run against the WGS databases or whole genome sequences. Multiple sequence alignments were performed and phylogenetic trees were constructed with ClustalW[51] using default parameters.

The three dimensional structures of CEACAM N domains were modeled using the Geno3D-release 2 software based on the published crystal structure of the murine CEACAM1 N-A2 fragment[37,52]. Leader and transmembrane domains were identified with the aid of programs SignalP and TMHMM, respectively. The NetPhos 2.0 server was used to produce neural network predictions for serine, threonine and tyrosine phosphorylation sites in the cytoplasmic domains of canine CEACAMs[53]. Comparison of the number of nonsynonymous substitutions per nonsynonymous site (Ka) with the number of synonymous substitutions per synonymous site (Ks) was performed with the Ka/Ks Calculation Tool [54]. The substitution rate ratio Ka/Ks measures the molecular selective pressure. If Ka/Ks = 1, the amino acid changes are neutral and will be fixed at the same rate as silent mutations. If Ka/Ks < 1, the amino acid changes are deleterious and purifying selection will reduce the fixation rate. If Ka/Ks > 1, the amino acid changes are evolutionarily advantageous and positive selection will increase the fixation rate.

## Abbreviations

CEA- Carcinoembryonic antigen; 

CEACAM- Carcinoembryonic antigen-related cell adhesion molecule; 

Cyt- Cytoplasmic domain; 

GPI- Glycosylphosphatidylinositol; 

TM- Transmembrane domain.

## Authors' contributions

RK conceived the study and did most of the biocomputing, TP cloned all cDNAs and performed RT-PCR analysis, SH collected and prepared mRNAs from tissues and cells, BBS and WZ participated in biocomputing and writing the manuscript. All authors participated in the design of the study, and they all read and approved the final version.

## Supplementary Material

Additional file 1 Full length cDNA sequences of canine CEACAM16, CEACAM18, CEACAM19, CEACAM20. The data provided represent the predicted cDNA sequences of canine CEACAM16, CEACAM18, CEACAM19, CEACAM20. Click here for file
