# Supplementary material for: Species-specific evolution of immune receptor tyrosine based activation motif-containing CEACAM1-related immune receptors in the dog
Source: BMC Evol Biol. 2007 Oct 18;7:196. doi: 10.1186/1471-2148-7-196 (PMC2110893; doi:10.1186/1471-2148-7-196)
Supplement: Additional file 1 — Full length cDNA sequences of canine CEACAM16, CEACAM18, CEACAM19, CEACAM20. The data provided represent the predicted cDNA sequences of canine CEACAM16, CEACAM18, CEACAM19, CEACAM20. [file 1471-2148-7-196-S1.doc]

**Additional file**

Full length cDNA sequences of canine CEACAM16, CEACAM18, CEACAM19, CEACAM20

5´- and 3´-untranslated regions are shown in green, sequences corresponding to exons in blue and black letters. Amino acid sequences are depicted in the one letter code. Asparagines within potential N-glycosylation sites are shown as bold letters. Differences in prediction are shown in red.

CEACAM16 tccaaatcaccgaaccctcccaggtcccgcgccagacagagccaggtccccaccaggaagggcctcggagcactgggacgccaccaccgccgtcttcaagaccaagttgggggcgaaagATGGCTGTGACCGGGTGCAGCTGGCTCCTCCTCAGTGCCACTTTTCTGAGCGTGGGGGCCGAGATCTCCATCACCCCCGAGCCCGCCCAGCCAGCTGAGGGGGACAACGTGACGCTGGCCGTCCACGGGCTTTTGGGGGAGCTGCTTGCCTACAACTGGTACGCGGGACCCACTCTCAGCCTGACTTACCTGGTGGCCAGCTACATTGTGAGCACAGGCGACGAGACCCCTGGCCCGGCCCACACAGGGCGGGAGGCTGTGCGCCCCGATGGCAGCCTGGACATCCAGGGCGTCCTGCCCCGCCACTCGGGCACCTACATCCTGCAGACTCTCAACAGGCAGCTGCAGACGGAGGTGGGCTACGGACACTTGCAGGTCTATGAGATCCTGGCCCAGCCCGTGGTCATGGCCAACAACACGGCACTGGTGGAGCGCCGAGACACCCTACGCCTGATGTGCAGCAGCCCCAGCCCCGCTGAGGTCCGCTGGTTCTTCAACGGCGAAGCCCTGCCCATCGCCATCCGCCTTGGCCAGTCCCCCGACGGCCGGGTGCTGACCCGGCATGGCATCCGCAGGGAGGAGGCTGGAGCCTACCAGTGTGAGGTCTGGAACCCGGTCAGTGTGAGCCGCAGCGAGCCCATCAACCTGACCGTGTACTTTGGCCCAGAACGCGTGGCCATCCTCCAGGATTCTACTGCCCGCACAGGCTGTACCATCAAGCTTGACTTCAATACATCCCTCACCCTGTGGTGTGTGTCCCAGTCCTGCCCAGAGCCCGAGTACGTGTGGGCCTTCAACGGGCGTGCCCTCAAGAACGGGCGAGACCACCTCAACATCAGTAGCATGACAGCAGCCCAGGAGGGCACGTACACGTGTATTGCTAAGAACCCCAAGACCCTGCTTTCCGGATCTGCCTCAGTGGTGGTCAAGCTCACCGCGGCAGCAGTTGCCATGACAATTGTGCCTGTGCCGACCAAGCCGATGGAGGGCCAGGACGTGACACTGACCGTACAGGGCTACCCCAAGGACCTGCTGGTCTATGCCTGGTACCGCGGGCCTGCCTCCGAGCCCAACCGGCTGCTCAGCCAACTGCCGTCAGGGAACTGGATCGCAGGCCCCGCACACACAGGCCGGGAAGTGGGCTTCGCCAACTGCTCACTGCTGGTGCAGAAGCTGAACCTCACGGATGCTGGCCGCTACATGCTCAAGACCGTCACATTGCAGGGCAAGACAGAGACGCTGGAAGTGGAGCTGCAGGTGGCCCCCCTGGAGTAGcagcacagctgtgaccgtggggacctccggagagaagaactcttcacaccgtcctccgtcctccccgtctcctccctct

MAVTGCSWLLLSATFLSVGAEISITPEPAQPAEGD**N**VTLAVHGLLGELLAYNWYAGPTLSLTYLVASYIVSTGDETPGPAHTGREAVRPDGSLDIQGVLPRHSGTYILQTLNRQLQTEVGYGHLQVYEILAQPVVMA**N**NTALVERRDTLRLMCSSPSPAEVRWFFNGEALPIAIRLGQSPDGRVLTRHGIRREEAGAYQCEVWNPVSVSRSEPI**N**LTVYFGPERVAILQDSTARTGCTIKLDF**N**TSLTLWCVSQSCPEPEYVWAFNGRALKNGRDHL**N**ISSMTAAQEGTYTCIAKNPKTLLSGSASVVVKLTAAAVAMTIVPVPTKPMEGQDVTLTVQGYPKDLLVYAWYRGPASEPNRLLSQLPSGNWIAGPAHTGREVGFA**N**CSLLVQKL**N**LTDAGRYMLKTVTLQGKTETLEVELQVAPLE

CEACAM18

ATGGATCCTTCCAGACCCAGATGCAGACTATGGAGGAAGATGGTTCTCCTAGCCAGTCTGCTGGCCTGTGGGATCCACCAAGCCTTTGGCCAAATGTTCATCAGCCCAGACTCACTTATAGGAGTCAAGGGATTTCGGACTGTCCTGGTCCTCGAGAATGCCACCCAAGATGCTCAGGAATACAGTTGGCACCGTGGTGCAGAGGACACTGTGGAAAATATGATTGTCAGCTACAAACCTCCCTTCAATTCCTGGCTATCTGGGCCTATGTTCAGCGGCCGGGAGAATGTGACCAGGTTGGGTGACCTGGTGATCAGGAGATCTGCATTTAGTGACACAGGGAACTACACTGTAAGGGTGGACACAGGCAATGAGACCCAGAGAGCAACTGGCTGGCTTGAAATTCAAGAGTTGGAAAGCAAACCAGAGATCTGGGCCAACACCAGCTCTGTGGTAGAGGACGTGGATTCTGTGGCTGCCATTTGCTACACCAATGCCACCAATATCAGGTGGTACGTGGATTACACACTGGTATCCAGCAATGACCGGATGACAATCTCCCCGGACCTCCAGACCCTCATCATCCACAGGGTCACCCGCCGAGACAGAGCACTTTATTGTGAGATAGATACTATCATGGAGATTCCTCGGAGGAGTGAAATTCTCTCTCTAACTGTGGCCTATGGGCCAGATGAAGTGTTGCTGAGTACCAGTCCCAGTATCTTCAAAGGTGTCCTATCTGCTAAGATAGGCTCCCAGGTGGATATGGCGTGTACTGCCTTTTCTGTTCCAAGTCCCAAGTATCATTGGAGCCACAATGGCTCTCTCCTAAGCTTCTCAGATGCAAGCATCACTCTCCCAAGTCTGGCCTGGGAACAGATGGGCAGATACAGATGCATCGTGGACAACCCCGTGACCCAGCTGACGATGTACAGAGAATTCCAGATCCAGACACCCCGGAACATTCCTGTTGTTGTAAACAGAGGTTTCTACATCTCAGGAGCCAAAGTGGTGTGGCTCATTGTGATGATAGTCCTGGGCAGCCTCTACATCTGTGGAATCCTGATCTACGGCTTGATCAGCAATTTATCCATCAGGCGGAGCCAGTTAAATGAGTGTTAA

MDPSRPRCRLWRKMVLLASLLACGIHQAFGQMFISPDSLIGVKGFRTVLVLENATQDAQEYSWHRGAEDTVENMIVSYKPPFNSWLSGPMFSGRENVTRLGDLVIRRSAFSDTGNYTVRVDTGNETQRATGWLEIQELESKPEIWANTSSVVEDVDSVAAICYTNATNIRWYVDYTLVSSNDRMTISPDLQTLIIHRVTRRDRALYCEIDTIMEIPRRSEILSLTVAYGPDEVLLSTSPSIFKGVLSAKIGSQVDMACTAFSVPSPKYHWSHNGSLLSFSDASITLPSLAWEQMGRYRCIVDNPVTQLTMYREFQIQTPRNIPVVVNRGFYISGAKVVWLIVMIVLGSLYICGILIYGLISNLSIRRSQLNEC

CEACAM19

tgtctgcagtggccctttggcacctccacaagacaccaagATGGAGATTCCTGAGTGGGCCCAGCACTACTTCTCAAAGGGCCTCCTGCTCTCAGCCTCAATCCTGGCCCTCTGGATCCCACAAGGCTCTTGGGCTGCCCTACGCATCCAGAAGATTCCAGAGTATCCTCAAAAAGACCAGGACCTTCTCCTGTCTGTCCAGGGCATCCCAGGCAACTTTCAGGACTTCAACTGGTACCTGGGGGAGGAGACCAATGGTGGCACGATGTTATTCACCTACTTCCCCGATCTCCAGTGGCCCCAGAGGGACGGCAGTGCCATGGGACAGCATGACATCGTTGGCTTCCCCAATGGCTCCATGATGCTGCATCGTGTCCAGCCCACCGACAGTGGCACCTACCAGGTAGCTGTCAACATCAATCCTGCCTGGATCATGAGGGCCAAGACTGAGGTCCAGGTGGCCGAAAAGCATAAGGAGCTGCCCATCATACACCTGCCCGTGAGTGCTGGGATCATGGCTGCCATCATAATTGGGTCCCTCTCTGCCGGGTCCCTCTTCATCTGCTGCATTGCCCATCTCCTGTTAACAGGAGGCTGGAGGGGCCAGAGCCACAGGATGACACCCACAGAGAAGCCAGAGGCGCGCCCCAACCTCAATGCTGGTGACCAGCACATCTATGAGGTGATGCCGTCTCCGACCCACCTGGTGTCTCCCCTCGGGGGCACAGCGTCCATGAACAACACCATGCCCCTGCCCCAGCAGCAGCCGGAGCCAGAGAACCACCCCTACCAGGACCTGCTGAACCCCGACCCTGACCCCTACTGCCAGCTCACGCCAACCCACTGAagaggtcccaggcccctg

MEIPEWAQHYFSKGLLLSASILALWIPQGSWAALRIQKIPEYPQKDQDLLLSVQGIPGNFQDFNWYLGEETNGGTMLFTYFPDLQWPQRDGSAMGQHDIVGFP**N**GSMMLHRVQPTDSGTYQVAVNINPAWIMRAKTEVQVAEKHKELPIIHLPVSAGIMAAIIIGSLSAGSLFICCIAHLLLTGGWRGQSHRMTPTEKPEARPNLNAGDQHIYEVMPSPTHLVSPLGGTASMNNTMPLPQQQPEPENHPYQDLLNPDPDPYCQLTPTH

CEACAM20

ctgaagggatagagctggagtgaagacagtgtctgtgtgtgcaagccagggcacgctggtcaggtgccaggacccATGGGGCTCGCTGACTTGTGGGGCCACCACTGGGTGGGAATCCTGCTTGCAGCCTCACTTTTGACCATGTGGAGTCTACCAGCTGCAGCCCAACTCACCCTTGATACCAACCCATTTACCACCACCCAAGGTGAGAAGGATGCTGTTCCATCTATGTCTGGGACCCCCTGGGCAACTCAGACTCATGGCAGATTCATAGATGTGGACACAGAAAGCAAAGCTATCCTTCTGATCCCTGCCTTCATACCTAAATCACCATTAGGATTCCATACTCGAAGACTGCTGGCCAAGCCCACCATTTCAATCAGCCAAAACACTGCCACAGAGCAGATGGAACAGGTGACCTTCTACTGTTACACCAAGGATGCCAATGTTACCATCCAGTGGTTCTTCAAAGATGTCCCCCTGGTGTTCCATGAACGCATGCAGCTGTCCACGGATGGCAAGAACCTCACTATCCTCACTGTGCAACGGGAGGACTCTGGGATTTACAAATGTGAAGCTCAGGGTTTCCTCCATGTCCACAGGAGTGATCCCACCTTCCTGACTGTGAACTATGGTCCTGACCCATTTGAAATCAAGTTGGAGTCTGGCATATCCAGCGGGGAGGTGGTTGAGGTGATAGAGGGTTCCACTGTGACCCTCTCGGTGGACACACGGTCTCATCCACCTCCTGACTATTCCTGGTTTCTCCTCAACAACTCTCTCCCATCTTCCTCGATGAGAACATTTACCATCCAGGCTGTGTCCAAGGAGCATGAAGGCATGTACAGGTGTTTGGTGTCCAACATTGCCACCCACCTGCTCCGCCTGAGTGCTCTTGAAGTCCGAGTCCTTGAAATGCTGACTGAGCCTCACATTGTGCCCCCAACCCTGAATCTCATGGAAAATGCCAGCTCTGTGACCCTGACCTGCCAGACCTCCCACAAGGAGGCTGGAGTCCTGTGGTTCCTGAGGGGCCAGGCCCTCCTGCCCAACAAACACCTGGTGCTGTCGGCCAACAACAGGAGCCTGGTAATCCATGGCCTCCGGAGAGACGACACGGGACCCTATGAGTGTGAGGTCTGGAATTGGGGCAGCCGGGCACGAAGCAAGGCCGTAAGGCTCACCATCAGCTATGGCCCTGATCAAGTGGACATCACCAGAGGGGCAGCATCTGGGGCGGTCAGCACTGTCAAGGCAGAGCTCAACTCTAGCCTGACCCTGTACTGTCAGGCAGAGTCTCAGCCAGGCGCTGAGTTTCAATGGACCCTTGAACACTCCACCACTGTGTATATGGGGCAGCAGTTAATCATCGGGACCCTGACCTGGGAACACCAGGGTGTCTACAACTGCATGGCCTCCAACCCACTGACCCAGCTGGCCCGCTCTGCCTCGGTCCTGGTCACAGTGGTAGGTCCCAGGTCTTCCCTGTCTGCAAGAGTCATTGTTGGCATTGCCCTCGGGATCCTGATTTTCATTGCCCTGACTGCAGGCCTGGGCTATTTCCTCTACAACAGAAATGCCAAACGGTTCTCAAGGAAAAAAGCAGAGGACCCCGTCCAGGAGGCAGCAACACCCAGCTCTGCGGAGGAGCCCTGTGCAGAGTCCTGTTCTAATTGGCCAAAGGCCATGTATGCCAATTTACCTGAACCTCAAGGACAAGTTGGAGTCAAAGAGATGCTGCCACCAGATCCCCTAGAGCAATTTTATGAGAAGGATCCACCATCAACAGCCTCTGGATACTATTGCCATGGCCCCAGAAAACCATCTTCCACAGTTGCATTGGATCCACTGGTCCCCACTCTACCAAAAGGAAACACAGAGTCAGACTATGAGGTGCTTGTGAATCCAGAACAGAACATTTACTGCCAGATCAACCCCTCAGTCTAAcagaagcagatttccttcagaaatcctagaaaaatcacgctcaatcagatattcaatgaaatgtattgggttcctatt

MGLADLWGHHWVGILLAASLLTMWSLPAAAQLTLDTNPFTTTQGEKDAVPSMSGTPWATQTHGRFIDVDTESKAILLIPAFIPKSPLGFHTRRLLAKPTISISQNTATEQMEQVTFYCYTKDA**N**VTIQWFFKDVPLVFHERMQLSTDGK**N**LTILTVQREDSGIYKCEAQGFLHVHRSDPTFLTVNYGPDPFEIKLESGISSGEVVEVIEGSTVTLSVDTRSHPPPDYSWFLL**N**NSLPSSSMRTFTIQAVSKEHEGMYRCLVSNIATHLLRLSALEVRVLEMLTEPHIVPPTLNLME**N**ASSVTLTCQTSHKEAGVLWFLRGQALLPNKHLVLSAN**N**RSLVIHGLRRDDTGPYECEVWNWGSRARSKAVRLTISYGPDQVDITRGAASGAVSTVKAEL**N**SSLTLYCQAESQPGAEFQWTLEHSTTVYMGQQLIIGTLTWEHQGVYNCMASNPLTQLARSASVLVTVVGPRSSLSARVIVGIALGILIFIALTAGLGYFLYNRNAKRFSRKKAEDPVQEAATPSSAEEPCAESCSNWPKAMYANLPEPQGQVGVKEMLPPDPLEQFYEKDPPSTASGYYCHGPRKPSSTVALDPLVPTLPKGN

TESDYEVLVNPEQNIYCQINPSV

Our CEACAM16 prediction is identical to: >gi|73948199|ref|XM_541572.2| PREDICTED: Canis familiaris chromosome 1 GSS clone AHTH152 (LOC484457), mRNA.

The CEACAM18 prediction is partially identical to: >gi|73947838|ref|XM_541462.2| PREDICTED: Canis familiaris similar to CEA-related cell adhesion molecule 1 (LOC484346), mRNA

Our CEACAM19 prediction assumes a sequencing error at position 86 (one G deletion) of the coding sequence ([ENSCAFG00000004663](http://www.ensembl.org/Canis_familiaris/geneview?gene=ENSCAFG00000004663;db=core)).

The CEACAM20 prediction is partially identical to: >gi|73948207|ref|XM_533647.2| PREDICTED: Canis familiaris similar to CEA-related cell adhesion molecule 20 (LOC476441), mRNA
